# A novel 3D imaging system for strawberry phenotyping

**DOI:** 10.1186/s13007-017-0243-x

**Published:** 2017-11-08

**Authors:** Joe Q. He, Richard J. Harrison, Bo Li

**Affiliations:** 1 NIAB EMR, New Road, East Malling, ME19 6BJ UK; 2 University of Reading, Whiteknights, Reading, RG6 6AH UK

**Keywords:** 3D imaging, Multi-view stereo, Point cloud analysis, High-throughput phenotyping

## Abstract

**Background:**

Accurate and quantitative phenotypic data in plant breeding programmes is vital in breeding to assess the performance of genotypes and to make selections. Traditional strawberry phenotyping relies on the human eye to assess most external fruit quality attributes, which is time-consuming and subjective. 3D imaging is a promising high-throughput technique that allows multiple external fruit quality attributes to be measured simultaneously.

**Results:**

A low cost multi-view stereo (MVS) imaging system was developed, which captured data from 360° around a target strawberry fruit. A 3D point cloud of the sample was derived and analysed with custom-developed software to estimate berry height, length, width, volume, calyx size, colour and achene number. Analysis of these traits in 100 fruits showed good concordance with manual assessment methods.

**Conclusion:**

This study demonstrates the feasibility of an MVS based 3D imaging system for the rapid and quantitative phenotyping of seven agronomically important external strawberry traits. With further improvement, this method could be applied in strawberry breeding programmes as a cost effective phenotyping technique.

## Background

A successful strawberry breeding programme generates and selects genotypes with traits suitable for the industry in its target geographic region [1]. As often genotypes cannot be directly observed, traditional breeding selects on the basis of a weighted selection index of phenotypes [2]. In order to maximise the accuracy of selection, heritable traits of interest must be measured precisely and accurately. Currently, most external fruit quality phenotyping approaches in strawberry breeding relies on the human eye to make assessments [1]. This approach is labour-intensive, prone to human bias and typically generates ordinal data less suitable for the most powerful quantitative statistical models [3].

Use of image analysis has the potential to overcome some of these limitations, with previous studies showing success in utilising 2D high-throughput imaging systems to assess external fruit quality [4]. Most studies were focussed on colour analysis of fruits, including apple [5], citrus [6], mango [7] and banana [8], but some systems have assessed morphological attributes, including the size of apples [9] and the shape of oranges [10]. For strawberry, an automated grading system was developed by Liming et al. [11] that assesses colour, size and four degrees of shape. In another 2D strawberry imaging system, developed by Nagata et al., the maximum fruit diameter could be derived by automatically identifying the axis from the top of the calyx to the tip of the nose [12]. However, 2D image analysis is not always a reliable fruit phenotyping method due to uneven colour distribution of fruit and occlusion of morphology from different viewing perspectives [13].

Recently, 3D imaging has been increasingly explored, as the cost of hardware decreases and reassembly techniques improve [14], with a range of sensors deployed for plant phenotyping. Light detection and ranging (LiDAR) was used to generate detailed 3D models of plants [15, 16], but is currently expensive, time-consuming and complex to implement [17]. Binocular stereovision is a low-cost solution for 3D plant canopy reconstruction [18], but with only two viewing perspectives, is insufficient to model the entire target. Other techniques, including time-of-flight (TOF) [19, 20] and structured light [21], have similar limitations in gathering 360° information from the target.

Studies of 3D imaging based phenotyping of fruit are limited. A 3D model of mango was generated using four cameras and the shape-from-silhouettes reconstruction method, but it did not encompass 360° of the fruit. Five parameters were extracted from the 3D model including length, width, thickness, volume and surface area in order to sort the mangoes by size. Image based sorting accuracy was comparable to manual sorting, but no comparison of individual trait data to a “gold standard” was shown [22]. Shape-from-silhouettes was also successfully applied to the 3D reconstruction of tomato seedlings with ten calibrated cameras. Stem height and leaf area were accurately measured after geometry based segmentation [23].

**Fig. 1:**
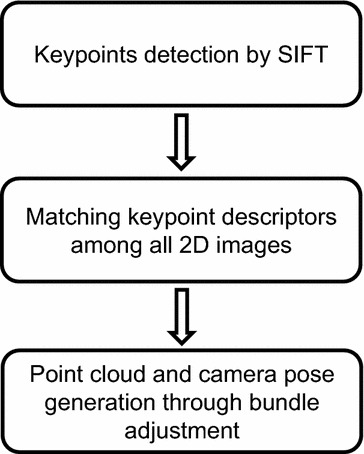
The flowchart of SfM method

Multi-view stereovision (MVS), which originated from binocular stereovision, is a promising approach for fruit phenotyping by capturing images from multiple overlapping viewpoints [24]. For the determination of the intrinsic camera parameters and the positions of uncalibrated cameras, Structure from Motion (SfM) is a widely used technique (Fig. 1) [25]. SfM detects feature points, called keypoints, from all the input 2D images using the Scale-invariant Feature Transform (SIFT) algorithm. The number of keypoints is determined by image quality, including factors such as resolution and texture. The relative pose and camera locations are determined by matching keypoints across all images and iteratively refined by bundle adjustment, resulting in a point cloud [24]. The coordinate system generated by SfM is always in an arbitrary image space making it necessary to transform the coordinate system into an object space by using a known standard [26]. This method has been demonstrated to be low cost, highly precise, easy to implement, generate 360° colour information, and require no camera calibration. MVS and SfM have been successfully utilised to generate estimates of leaf and stem dimensions of paprika [17].

In this study, a novel 3D imaging based approach for phenotyping strawberry fruit was explored. MVS and SfM were applied to generate a 3D model of strawberry and software was developed to measure seven agronomically important external strawberry traits. This method is promising to facilitate strawberry breeding by providing a high-throughput, objective and low-cost phenotyping system.

## Methods

### Fruit material

100 strawberries were purchased from local supermarkets, including 10 different varieties, to represent the diverse range of commercially available strawberry phenotypes. All fruits were assessed before their “best before” dates. Fruits would likely have been subjected to chilling to 4 °C within 4 h of harvest and kept at that temperature throughout the supply chain until sale. Fruits were stored at 4 °C until assessment.

### Manual assessment

In order to validate the results of the 3D analysis, phenotypic data were collected manually (Table 1) immediately after imaging. Measurements of dimensions were performed using a pair of digital callipers and measurement of volume was performed using an overflow can and a measuring cylinder.

**Table 1:** Manual scoring metrics for seven external fruit quality traits

External quality parameter	Scoring metric
Achene number	Number of achenes visible, without disturbing calyx
Calyx size	Maximum Euclidean distance between any pair of points on the calyx
Colour	Scale 1–8 (strawberry colour chart for experimental ends, Ctifl, France)
Height	Dimension of fruit from centre of calyx to tip of nose
Length	Greatest dimension of fruit orthogonal to the height
Width	Greatest dimension of the fruit orthogonal to both height and length
Volume	Volume of displaced water when fruit was completely submerged

### Image capture

**Fig. 2:**
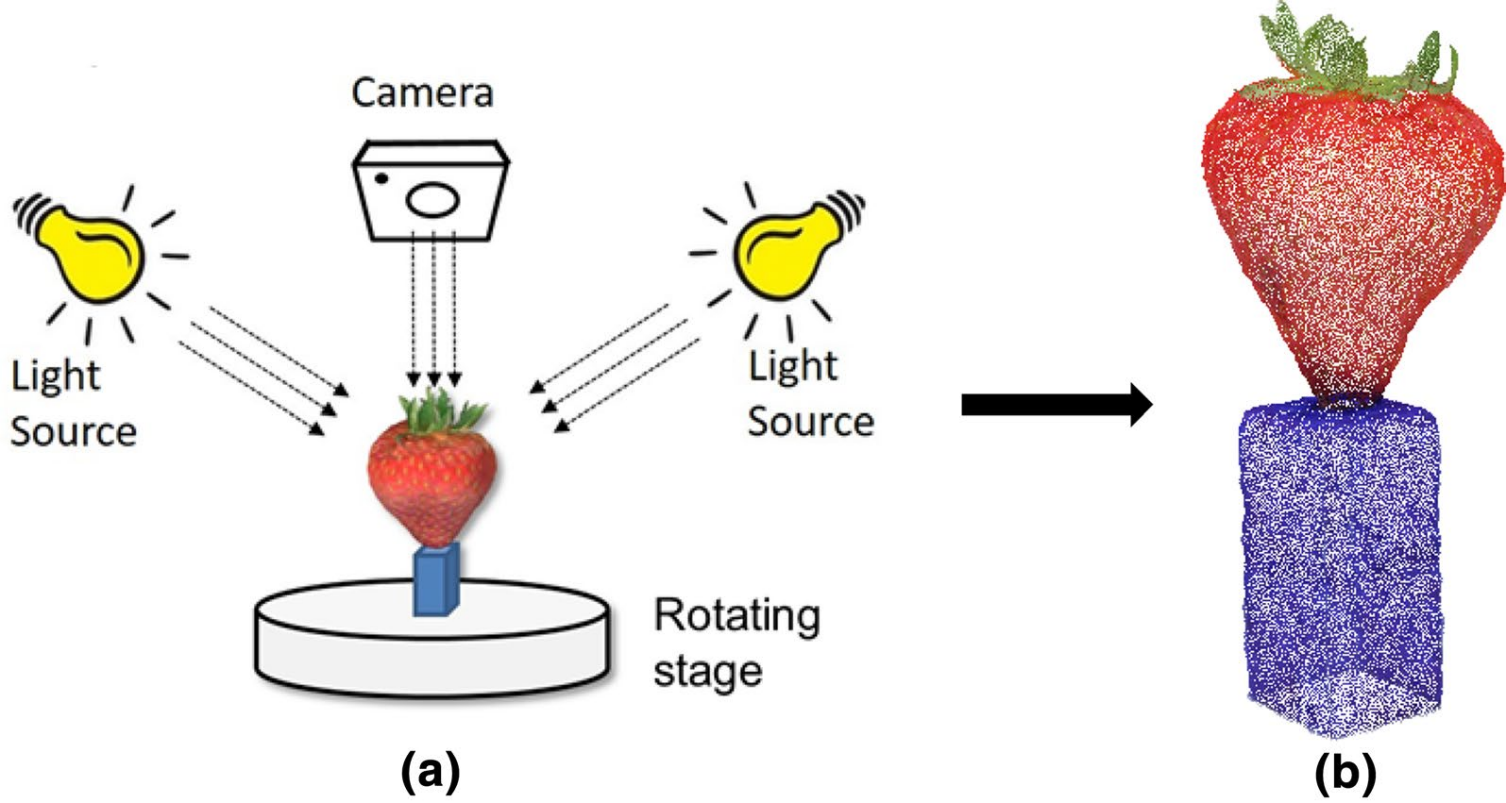
**a** Mechanical structure of the proposed imaging system; **b** point cloud of the strawberry and holder

The sample was pinned onto a dark blue holder (38 mm × 19 mm × 19 mm) placed in the middle of a turntable and rotated at 0.02 Hz. A single lens reflex (SLR) camera (Canon EOS 1200D, Canon Inc., Tokyo, Japan) was placed facing the sample with a focal length of 55 mm so that the field of view is large enough to accommodate the largest strawberry sample. The distance between the lens and the sample was set to 50 cm with a viewing angle of 35° to the horizontal, which allows maximum visualization of the strawberry body without occlusion of the calyx. The relative positions of the camera and holder was fixed for all samples. The sample was illuminated with two white LED light sources against a white background (Fig. 2a). In total, 146 images were captured per sample over 50 s with ISO speed rating at 800, shutter speed at 1/125 s and aperture value at 5.38 EV. With this configuration, no blurring was found in any image.

### 3D point cloud reconstruction

A Dell desktop computer (CPU Xeon^®^ CPU X5560 @2.80 GHz x 16, Intel Co., Santa Clara, CA, USA) with a graphics card (Quadro K2200 GPU, NVIDIA Co., Santa Clara, CA, USA) operating on Linux Ubuntu 14.04 was used in this study for both software development and point cloud processing.

The point cloud reconstruction was implemented with commercial software (Agisoft Photoscan, Agisoft, LLC, St. Petersburg, Russia; licence required), utilising the Structure from Motion (SfM) algorithm [17] (Fig. 2b). Due to the high overlap between adjacent images and high resolution (5148 × 3456) of each image, pre-processing software was developed to automatically reduce the number of images by discarding three frames from every four. This was found to be the minimum number to reconstruct all the 3D models successfully. Additionally, each image was rescaled to the resolution of 1000 × 1450, which greatly increased the processing speed with satisfactory point cloud quality.

### Automated 3D image analysis

The automated point cloud analysis software was developed in C++ with Point Cloud Library (PCL) [27]. The software is programmed to automatically load all point cloud files in order and process them in a batch by implementing the point cloud segmentation and external quality attributes measurement algorithms.

#### Point cloud segmentation

**Fig. 3:**
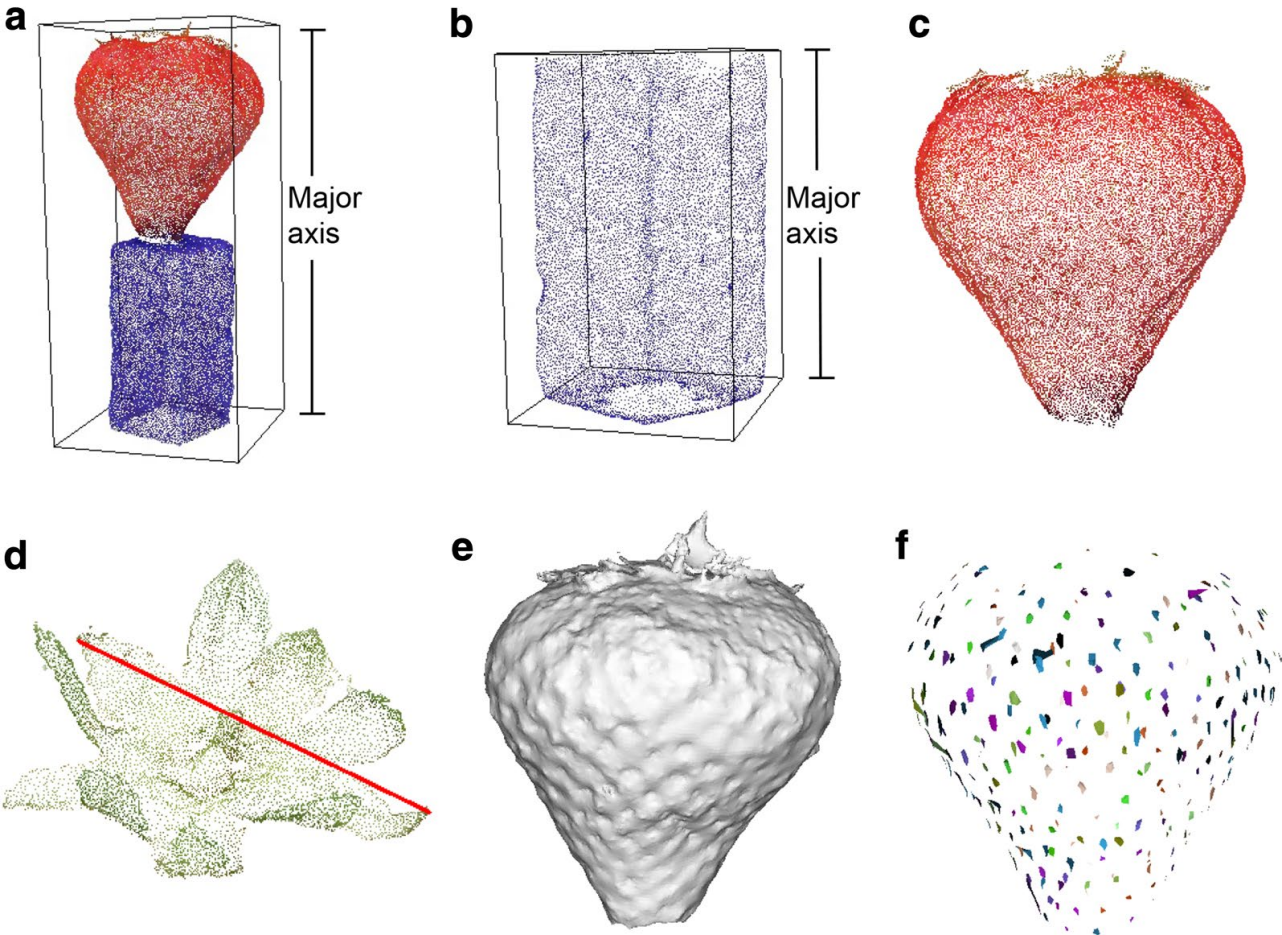
**a** Bounding box fitted on the point cloud strawberry body and holder; **b** Bounding box fitted on point cloud of holder; **c** Point cloud of strawberry body; **d** Point cloud of calyx with a red line to label maximum distance; **e** mesh of strawberry; **f** identification of achenes

Each point cloud was first converted from Red Green Blue (RGB) space to Hue Saturation Value (HSV) space. Using an arbitrary threshold on the hue channel, which is defined as the attribute of a visual sensation to one of the perceived colours [28], the point cloud was segmented into calyx, body, achenes and holder (Fig. 3a–d).

#### Orienting bounding box (oBB) fitting

The OBBs were fitted to the segments of holder and the combination the holder and the fruit body for the size measurement. The major eigenvectors of the covariance matrix of points in a point cloud define the major axis of its OBB [29]. The second axis was determined by calculating the maximum Euclidean distance of the points in the point cloud orthogonal to the major axis. The final axis was orthogonal to both other axes.

#### Height, length and width

An OBB was fitted to the point cloud of the combination of the fruit body and holder segments. The OBB was not fitted directly to the body, as its irregular shape often resulted in misidentification of the vertical axis. The height of the combination of fruit body and holder was always the largest dimension, so the magnitude of the OBB major axis was assumed to be equivalent to the height of the fruit body and holder model. As the fruit body was always longer and wider than the holder, the second and third dimensions of the OBB represented length and width respectively. The height of the holder was estimated by fitting an OBB to its point cloud and the difference in height between it and the combination of fruit body and holder OBB was assumed to be the height of the fruit. Ratios between the three fruit body dimensions and the height of the holder were multiplied by the true height of the holder to derive the strawberry height, width and length.

#### Volume

The mesh of the strawberry body (Fig. 3e) was constructed from the point cloud using Poisson Surface Reconstruction [30], which produces an enclosed mesh without any edges or large holes. The mesh volume was calculated by summing the volume of every triangle based pyramid formed from each face of the mesh and the origin of the point cloud [31].

#### Calyx size

The edges of the calyx segment were identified by applying convex hull [32], enabling rapid calculation of the maximum Euclidean distance (Fig. 2d). The ratio between the calyx maximum distance and the height of the holder OBB was multiplied by the true height of the holder to estimate calyx size.

#### Achene number

The segmentation of achenes from the point cloud was based on identifying points in the body segment with an arbitrary range in the hue channel of HSV space. A clustering algorithm based on Euclidean distances between points was implemented to group points corresponding to the same achene (Fig. 3f) [33] and the number of clusters was counted automatically.

#### Colour

As hue value in HSV space represents visual sensation of perceived colour [28], the mean intensity of the hue channel of every point in the body segment was calculated for the assessment of the strawberry colour.

### Statistics

In this study, the concordance correlation coefficient (CCC) was used to measure the concordance between the manually derived and the 3D image derived external fruit quality traits [34]. Additionally, the coefficient of determination (r^2^) was calculated to estimate correlation between the sets of values. Statistical analysis was performed using R [35]. Linear models and associated coefficients were derived using the “lm” function, the root mean square error (RMSE) was derived using the “Metrics” package [36] and the concordance correlation coefficient (CCC) was derived using the “Agreement” package [37].

## Results

In order to evaluate the measurement of seven external strawberry fruit quality parameters using 3D imaging (hereafter referred to as automated assessment), 100 berries were automatically and manually assessed. Reliable reconstruction could be achieved by taking a minimum of 37 images per berry with 100% successful reconstruction, though the nose of the fruit was often missing due to occlusion from the shooting angle. With the described setup, data capture took approximately 60 s, including 10 s of operator action per sample. Model reconstruction took approximately 15 min and parameter derivation took approximately 50 s. Both these operations were fully automated.

In order to validate the 3D reconstruction, the point cloud of the holder segment was manually measured in Meshlab [38], an open source software for 3D mesh visualisation. Although their absolute sizes in image space were inconsistent (range 0.36–1.73; mean 0.78; SD 0.27), ratios among the height, width and length were the similar to the true ratios. As there was no evidence of distortion, the absolute height of the holder was used as a standard for fruit dimension measurements. Moreover, incorporation of the holder point cloud ensured that the vertical axis was always greater than any other axis, allowing the major eigenvector of the point cloud covariance matrix to consistently define the vertical axis.

**Fig. 4:**
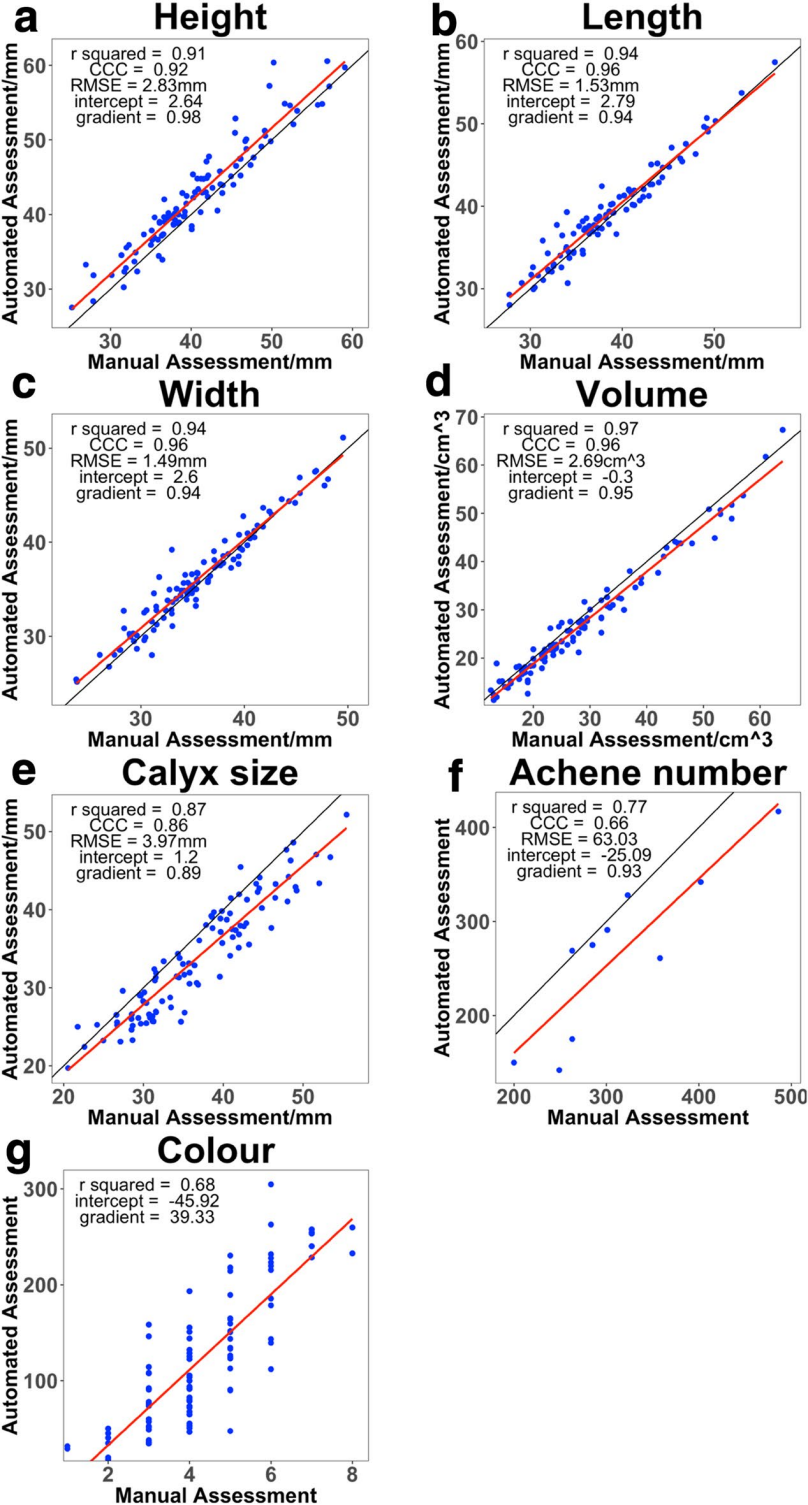
Regression analysis for height (**a**), length (**b**), width (**c**), volume (**d**), calyx size (**e**), achene number (**f**) and colour (**g**) as measured by automated assessment and manual assessment. Sample size = 100 for all measurements, except achene number, where sample size = 10. Red lines are least squares linear regression curves and black lines are idealized regression curves (y = x)

 To validate the measurements, the seven traits were measured on a sample of 100 fruits using both manual and automated assessment (Fig. 4). Concordance and correlation were assessed using CCC and r^2^ respectively. Good concordance (CCC > 0.9) and correlation (r^2^ > 0.9) were found between the measurements of fruit dimensions and volume (Fig. 4a–d). Weaker concordance (CCC = 0.86) and correlation (r^2^ = 0.87) was found between the measurements of calyx size (Fig. 4e), which was possibly due to the soft calyx being moved during assessment. Weak concordance (CCC = 0.67) and correlation (r^2^ = 0.77) were found between the measurements of achene number (Fig. 4f), which is possibly due to the lack of information gathered regarding the nose of the fruit. Weak correlation (r^2^ = 0.68) was found between the measurements of colour (Fig. 4g), with high variance in the manual scores. This was likely due to the variability of colour on each fruit and the subjective nature of the score.

## Discussion

Good concordance between manual and automated measurements of calyx size, height, length, width and volume, and promising results for achene number and colour were achieved. It is suggested that the qualitative traits of strawberry currently used in breeding can be represented using the measurements generated from this study. For instance, a “long conic” [1] fruit has a large ratio of height to width and measurement of “Cap size” [1] can be defined by the ratio of calyx size to fruit width and length.

With further development, automated assessment could be suitable for integration into existing strawberry breeding programmes, bringing a range of advantages. Firstly, the quantitative, accurate and unbiased measurements would increase the accuracy of the selection in strawberry breeding. The precise measurements would be particularly suitable for input into models of genomic selection, which attempt to quantify small effect quantitative trait loci (QTLs) associated with polygenic traits [39, 40]. Secondly, automated assessment has the potential to improve the speed of assessment. The described setup requires approximately 10 s of human operator time per sample, approximately 20-fold faster than making the equivalent manual measurements. Thirdly, the low cost and wide availability of hardware mean that this approach can be easily introduced into existing breeding programmes with minimal capital expenditure.

Measurement error may have arisen from a range of sources. During manual assessment, the axis of measurement was determined by eye, potentially resulting in non-maximal distances or non-orthogonal axes. As the calyx is soft, errors may have been induced in the operation of the callipers. Correlation between the measurements of colour may be weak as manual assessment is subjective and it is difficult to assess fruit with uneven colour distributions.

This imaging system can be developed to reduce the duration of data capture by using alternative imagers such as scientific cameras or webcams with programmable shutter speeds and resolutions. Reducing resolution to 1000 × 1450 greatly increases the processing speed compared to the original images, but further investigation is needed to identify the minimum resolution to generate satisfactory point clouds. Use of multiple calibrated cameras to capture information from different viewpoints simultaneously could also be explored to further improve the quality and efficiency of 3D reconstruction, particularly from the nose of the fruit and the data capture speed.

As both fruit body and achenes have a range of colours, our current algorithm of arbitrary hue thresholding is unlikely to be reliable in identifying achenes from a range of cultivars. More sophisticated adaptive or texture based thresholding algorithms would likely improve the cluster identification.

It is believed that more traits could be derived from the gathered dataset. Firstly, algorithms exist that can calculate the surface area of a 3D mesh [31], which together with reliable achene counts could be used to quantify achene density. Secondly, rotational symmetry could be quantified by considering the distribution of the Euclidean distance of points to the principal axis in 2D slices of the point cloud orthogonal to the principle axis. Thirdly, it may be possible to quantify the morphology of the fruit body at the neck of the fruit to derive information regarding the neck line.

## Conclusion

In this study, a MVS based 3D reconstruction pipeline was developed and utilised to generate in silico model of strawberries. Automated 3D image point cloud analysis software was developed in house to derive berry achene counts, calyx size, colour, height, length, width and volume of the model. This study found good correlation between the automated and manual assessment techniques for dimension measurements and volume, suggesting that automated assessment is a promising technique to be utilised in place of manual assessment for these traits.

The focus of this study was the investigation of the use of 3D imaging to phenotype strawberries for commercial breeding. This system, with further improvement, can be quantitative, accurate, rapid and require little capital investment to be integrated into existing strawberry breeding programmes. This approach can also be further developed for strawberry quality control as its high precision is particularly suited for assessing differences within single cultivars, a situation frequently encountered in pack houses.
